# (−)-Naringenin 4′,7-dimethyl Ether Isolated from *Nardostachys jatamansi* Relieves Pain through Inhibition of Multiple Channels

**DOI:** 10.3390/molecules27051735

**Published:** 2022-03-07

**Authors:** Ru-Rong Gu, Xian-Hua Meng, Yin Zhang, Hai-Yan Xu, Li Zhan, Zhao-Bing Gao, Jun-Li Yang, Yue-Ming Zheng

**Affiliations:** 1 School of Chinese Materia Medica, Nanjing University of Chinese Medicine, Nanjing 210023, China; 20190883@njucm.edu.cn; 2 Center for Neurological and Psychiatric Research and Drug Discovery, Shanghai Institute of Materia Medica, Chinese Academy of Sciences, Shanghai 201203, China; yinz1304613@163.com (Y.Z.); hyxu@simm.ac.cn (H.-Y.X.); zhanli@simm.ac.cn (L.Z.); 3 CAS Key Laboratory of Chemistry of Northwestern Plant Resources and Key Laboratory for Natural Medicine of Gansu Province, Lanzhou Institute of Chemical Physics, Chinese Academy of Sciences, Lanzhou 730000, China; mengxianhua@licp.cas.cn; 4 Zhongshan Institute of Drug Discovery, Institution for Drug Discovery Innovation, Chinese Academy of Sciences, Zhongshan 528400, China; 5 University of Chinese Academy of Sciences, No. 19A Yuquan Road, Beijing 100049, China

**Keywords:** (−)-Naringenin 4′,7-dimethyl ether, analgesic candidate, mechanism study, delayed rectifier potassium currents, ion channels

## Abstract

(−)-Naringenin 4′,7-dimethyl ether ((−)-NRG-DM) was isolated for the first time by our lab from *Nardostachys jatamansi* DC, a traditional medicinal plant frequently used to attenuate pain in Asia. As a natural derivative of analgesic, the current study was designed to test the potential analgesic activity of (−)-NRG-DM and its implicated mechanism. The analgesic activity of (−)-NRG-DM was assessed in a formalin-induced mouse inflammatory pain model and mustard oil-induced mouse colorectal pain model, in which the mice were intraperitoneally administrated with vehicle or (−)-NRG-DM (30 or 50 mg/kg) (*n* = 10 for each group). Our data showed that (−)-NRG-DM can dose dependently (30~50 mg/kg) relieve the pain behaviors. Notably, (−)-NRG-DM did not affect motor coordination in mice evaluated by the rotarod test, in which the animals were intraperitoneally injected with vehicle or (−)-NRG-DM (100, 200, or 400 mg/kg) (*n* = 10 for each group). In acutely isolated mouse dorsal root ganglion neurons, (−)-NRG-DM (1~30 μM) potently dampened the stimulated firing, reduced the action potential threshold and amplitude. In addition, the neuronal delayed rectifier potassium currents (I_K_) and voltage-gated sodium currents (I_Na_) were significantly suppressed. Consistently, (−)-NRG-DM dramatically inhibited heterologously expressed Kv2.1 and Nav1.8 channels which represent the major components of the endogenous I_K_ and I_Na_. A pharmacokinetic study revealed the plasma concentration of (−)-NRG-DM is around 7 µM, which was higher than the effective concentrations for the I_K_ and I_Na_. Taken together, our study showed that (−)-NRG-DM is a potential analgesic candidate with inhibition of multiple neuronal channels (mediating I_K_ and I_Na_).

## 1. Introduction

Pain is an unpleasant sensory and emotional experience associated with actual or potential tissue damage [1]. It can be subdivided into somatic pain and visceral pain according to the originating tissue, while it can be categorized into acute and chronic pain based on the ongoing time [2]. As a rising health problem, chronic pain is predicted to affect up to 30% of adults worldwide, and about 70% of patients are refractory to the current treatments [2,3]. The use of analgesics is one of the main therapies for the treatment of pain. Opioids, currently a mainstay of pain relief drugs, can cause adverse effects such as tolerance, dependence, opioid-use disorders as well as gastrointestinal dysfunction [4,5,6]. Huge unmet needs remain for patients with chronic inflammatory, musculoskeletal, visceral, and neuropathic pain conditions. There is an urgent need to identify novel non-opioid drugs and investigate the underlying mechanism, thereby benefiting the following structure-activity relationship studies.

The sensing and transmission of pain signals rely critically on the activities of ion channels expressed in afferent pain fibers, especially for the small-diameter dorsal root ganglia (DRG) neurons [7,8,9]. It is well established that voltage-gated sodium (Nav) and voltage-gated potassium (Kv) channels are responsible for determining the neuronal excitability, and genetic or pharmacological dysfunction of these channels was widely confirmed to cause pain in human and multiple animal models [10,11]. Among the nine reported Nav (Nav1.1~Nav1.9) isoforms, Nav1.7 and Nav1.8 channels are highly distributed in the peripheral nervous system (PNS) [12]. During an action potential, Nav1.7 is poised to help set a voltage threshold for action potential firing, and Nav1.8 contributes substantially to the rising phase of the action potentials in nociceptors during pain sensing [13]. Then, Kv channel-mediated potassium currents terminate the action potential by repolarizing the membrane potential [14,15]. The potassium currents in nociceptive DRG neurons consist of a rapidly inactivating component and a slowly inactivating component, which respectively correspond to the transient outward potassium currents (I_A_) and delayed rectifier potassium current (I_K_) [16,17]. The Kv2.1 channels are thought to represent a major component of I_K_, which helps to set the resting membrane potential and shape the action potentials [18,19,20]. Therefore, modulation of these channels underlies multiple analgesics.

**Figure 1:**
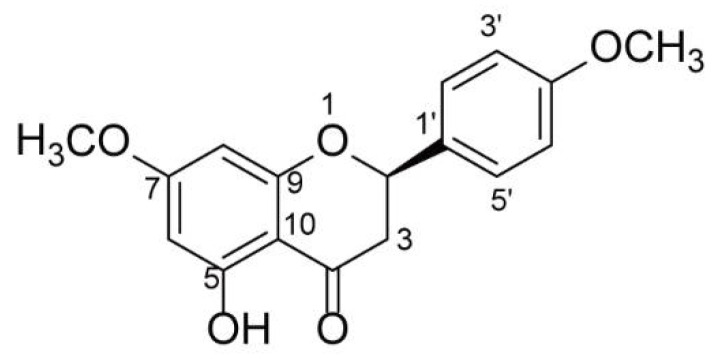
The structure of (−)-naringenin 4′,7-dimethyl ether ((−)-NRG-DM).

*Nardostachys jatamansi* has been widely used as a folk medicine in China, Nepal, Bhutan, India, and Japan for the treatment of pain, altitude sickness, fever, and wounds [21]. The roots of *N. jatamansi* have neuroprotective, sedative, and analgesic properties, and a variety of active ingredients have been separated from *N. jatamansi*, in which sesquiterpenoids, essential oils, iridoids, triterpenoids, flavonoid, coumarin, and lignin are the main chemical constituents [21,22,23]. The anti-inflammatory activities of sesquiterpenoids, terpenic coumarin, and phytosterol such as nardosinanones and narchinol might contribute to the analgesic activities produced by *N. jatamansi* [21,24,25]. Inhibition of the production of NO and inflammatory cytokines (IL-6, PEG2, TNF-a and IFN-a/β, etc.) and phosphorylation of MAPK signaling is implicated in the analgesic activity [25,26,27]. Flavonoids are polyphenolic structures naturally distributed in most plants and consumed daily. They have been widely used for analgesic, anti-inflammatory, and antioxidant effects along with safe preclinical and clinical profiles [28,29]. For example, troxerutin and quercetin act as anti-inflammatory agents that help reduce pain in clinics [30,31]. However, flavonoids with analgesic activities have not been reported from *N. jatamansi*. In the current study, a flavonoid named (−)-naringenin 4′,7-dimethyl ether ((−)-NRG-DM) was first isolated from *N. jatamansi* (Figure 1). As a flavonoid originating from a widely used folk medicine for pain relief, we hypothesized that (−)-NRG-DM might have analgesic activity similar to its naringenin prototype which has been reported to alleviate pain in multiple models [32]. To explore the hypothesis, the analgesic activity and the potential side effects of (−)-NRG-DM were examined in a formalin-induced mouse inflammatory pain model, mustard oil-induced mouse colorectal pain model, and mouse rotarod test. The underlying mechanism was studied in acutely isolated mouse small-diameter DRG neurons and heterologous expression cells using a standard whole-cell patch-clamp technique.

## 2. Results

### 2.1. Structure Elucidation of (−)-NRG-DM

(−)-NRG-DM was obtained as a white needle crystal, which was isolated from *N. jatamansi* by means of chromatographic methods, including HPLC and silica gel column chromatography. The structural assignments were confirmed by HRESIMS, ^1^H, and ^13^C NMR spectra, and compared with the literature data. The molecular formula was confirmed as C_17_H_16_O_5_ based on its HRESIMS ion at m/z 323.088 5 [M + Na]^+^ (calcd. 323.089 0) and 623.187 9 [2M + Na]^+^ (calcd. 623.188 8). The planar structure of (−)-NRG-DM was confirmed by the NMR data of (−)-NRG-DM, which were those of (+)-naringenin 4′,7-dimethyl ether [33,34]. (−)-NRG-DM has only one chiral carbon atom (C-2), and the specific rotation of (−)-NRG-DM was +7.8 (c 0.22 in CH_2_Cl_2_), which is contrary to that of sakuranetin [35]. Therefore, the absolute configuration of C-2 of (−)-NRG-DM was determined as R. Thus, the absolute configuration of (−)-NRG-DM was assigned as Figure 1.

### 2.2. Analgesic Effects of (−)-NRG-DM in Formalin-Induced Mouse Inflammatory Pain Model

**Figure 2:**
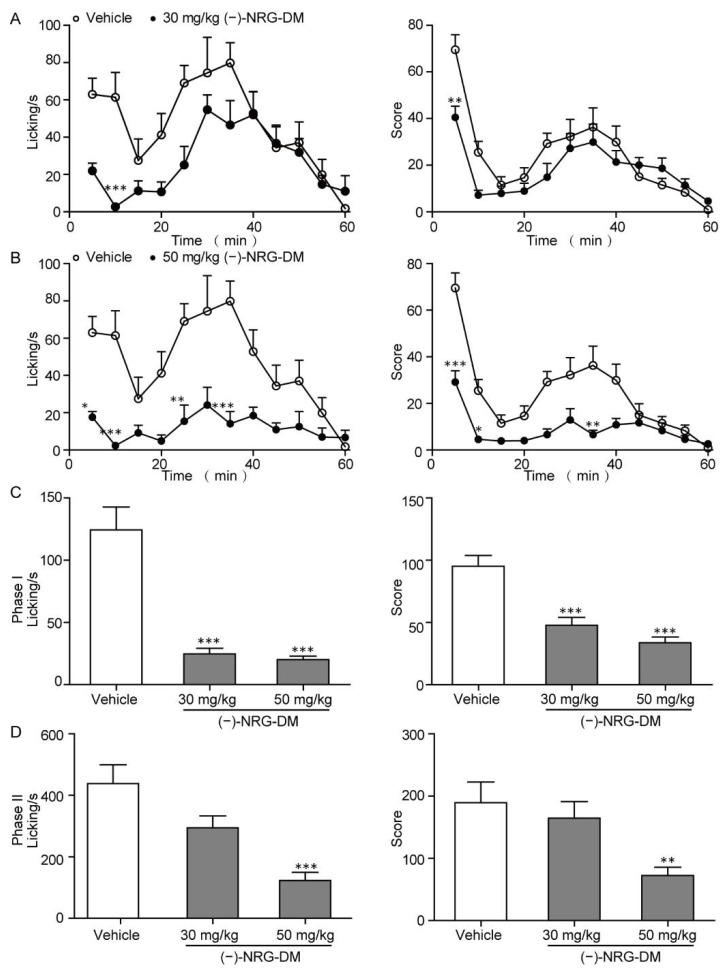
Analgesic effects of (−)-NRG-DM in the formalin-induced mouse inflammatory pain model. (**A**) 30 mg/kg compound (−)-NRG-DM attenuated the biphasic pain responses, including both licking time (left) and score (right) throughout the 60 min trial. (**B**) 50 mg/kg (−)-NRG-DM attenuated the biphasic pain responses, including both licking time (left) and score (right) throughout the 60 min trial. Bar graph showing the effects of vehicle (white), 30 mg/kg and 50 mg/kg compound (−)-NRG-DM (grey) on the pain behaviors during phase I (**C**) and phase II (**D**) in the formalin-induced mouse inflammatory pain model. In all groups, *n* = 10 animals. Statistical significance: * *p* ≤ 0.05, ** *p* ≤ 0.01, *** *p* ≤ 0.001.

The formalin test is a classical inflammatory pain model which represents somatic pain. Intraplantar injection of formalin results in a typical two-stage nociceptive behavior, which is characterized by licking and biting of the injected paw. The first phase (0~10 min) mainly reflects nociceptive pain, while the second phase (11~60 min) represents the inflammatory responses [36]. In our study, we tested the effects of (−)-NRG-DM on formalin-induced inflammatory pain in mice. (−)-NRG-DM at 30 mg/kg and 50 mg/kg body weight were individually intraperitoneally administrated 30 min before injection of 1% formalin solution. Consequently, (−)-NRG-DM significantly attenuated painful behaviors in a dose-dependent manner during phase I and phase II (Figure 2). At the dose of 50 mg/kg, (−)-NRG-DM significantly attenuated painful behaviors, including the licking time and overall pain score in both phases in formalin-injected mice. While at the dose of 30 mg/kg, (−)-NRG-DM shortened the licking time and pain score of the two behavioral stages, but only had a significant influence in phase I.

To exclude possible non-specific muscle relaxant or sedative effects, the effects of (−)-NRG-DM on motor performance were evaluated in the rotarod test. (−)-NRG-DM was well tolerated in the rotarod test, with no significant effects on the ability to remain on the rotating rod after intraperitoneal administration of (−)-NRG-DM at 100 mg/kg, 200 mg/kg, or even 400 mg/kg (Table 1). Together, these data showed that (−)-NRG-DM is a well-tolerated natural analgesic compound and can dose-dependently suppress somatic pain in vivo.

### 2.3. Analgesic Effects of (−)-NRG-DM in Mustard Oil-Induced Mouse Colorectal Pain Model

**Figure 3:**
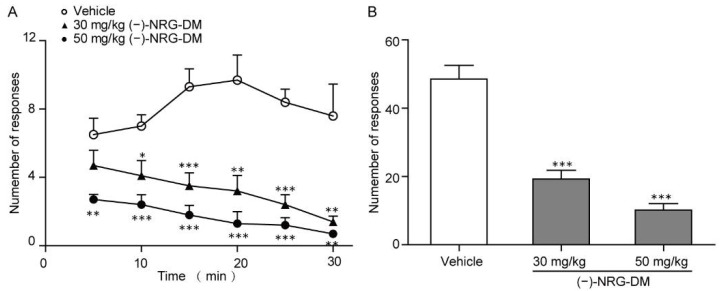
Analgesic effects of (−)-NRG-DM in the mustard oil-induced mouse colorectal pain model. (**A**) 30 mg/kg and 50 mg/kg (−)-NRG-DM attenuated the acute pain-related behaviors throughout the 30 min trial. (**B**) Bar graph showing the effects of vehicle (white), 30 mg/kg and 50 mg/kg (−)-NRG-DM (grey) on the writhing number caused by pain during a 30 min period in the mustard oil-induced mouse colorectal pain model. In all groups, *n* = 10 animals. Statistical significance: * *p* ≤ 0.05, ** *p* ≤ 0.01, *** *p* ≤ 0.001.

The dose-dependent relief of somatic pain in the formalin model prompted us to ask whether it could attenuate the visceral pain. Thereby, we constructed the mustard oil-induced mouse colorectal pain model as previously described [37]. After intracolonic application of 50 µL 0.75% mustard oil, the mice exhibited pain-related behavior (e.g., licking, stretching, squashing, or retraction of the abdomen) in the next 30 min observation period (Figure 3A). As those observed in intraplantar formalin-induced pain, (−)-NRG-DM dose dependently relieved intracolonic mustard oil-caused writhing (Figure 3A). After intraperitoneal application of 30 mg/kg and 50 mg/kg (−)-NRG-DM, the writhing number reduced by approximately 60% and 80%, respectively (Figure 3B). Our data showed that (−)-NRG-DM could attenuate visceral pain either.

### 2.4. Inhibitory Effects of (−)-NRG-DM on Action Potential Firing in Mouse DRG Neurons

**Figure 4:**
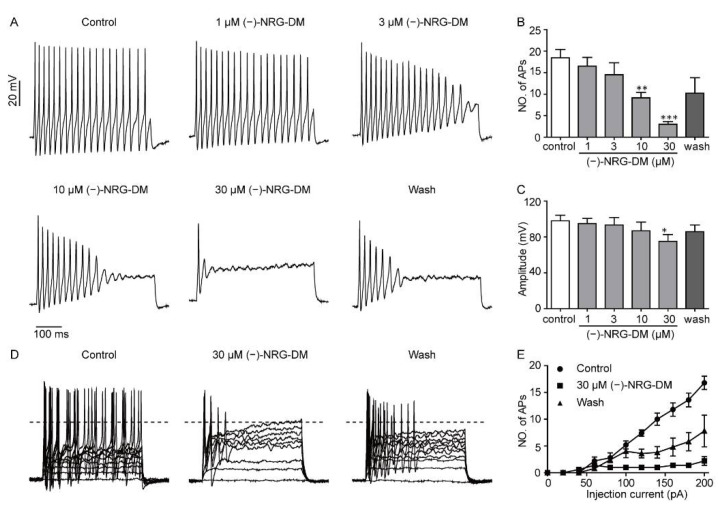
(−)-NRG-DM inhibited the neuronal excitability of acutely isolated mouse dorsal root ganglion neurons. (**A**) Representative traces of action potentials following 200 pA current injection with or without (−)-NRG-DM at indicated concentrations in DRG neurons. Bar graph showing the effects of (−)-NRG-DM on the firing frequency (**B**), amplitudes of the first action potential (**C**) before (control, white) and after the application of (−)-NRG-DM (grey) at indicated concentrations (*n* ≥ 5). (**D**) Responses of representative DRG neurons with or without 30 µM (−)-NRG-DM to 500 ms depolarization current steps for the generation of all-or-none action potential. (**E**) The averaged number of action potentials of DRG neurons before and after application of 30 µM (−)-NRG-DM (*n* = 5). Statistical significance: * *p* ≤ 0.05, ** *p* ≤ 0.01, *** *p* ≤ 0.001.

To investigate the underlying mechanism of (−)-NRG-DM-mediated analgesic activity, whole-cell current-clamp technology was applied to examine the effects of (−)-NRG-DM on the action potential firing in acutely isolated mouse small-diameter DRG neurons. The action potentials were evoked by a current injection of 200 pA for a 500 ms period. The threshold was defined by the first amplitude at which an action potential with a membrane potential larger than 0 mV was produced, and the amplitude was defined as the peak relative to the resting membrane potential [38]. Consistent with its analgesic activities in vivo, (−)-NRG-DM dose-dependently inhibited the firing frequency of the DRG neurons (Figure 4A,B). The amplitudes were reduced from 98.00 ± 6.19 mV to 95.00 ± 5.65 mV, 93.40 ± 8.27 mV, 86.80 ± 9.75 mV, and 74.40 ± 7.08 mV after perfusion of 1 μM, 3 μM, 10 μM, and 30 μM (−)-NRG-DM, respectively (Figure 4C, *n* = 5). Current threshold, the injection current required to elicit a single all-or-none action potential, was determined by applying 500 ms depolarizing currents of increasing magnitude. Surprisingly, the threshold of action potential firing was gradually reduced as the concentration of (−)-NRG-DM increased (Figure 4D, E, *n* = 5). The inhibitory effects of (−)-NRG-DM on the firing frequency and the amplitudes of action potentials were partially reversible after washout. These data indicated that (−)-NRG-DM can dampen action potential discharges in nociceptive DRG neurons.

### 2.5. Inhibitory Effects of (−)-NRG-DM on Neuronal Potassium Currents

**Figure 5:**
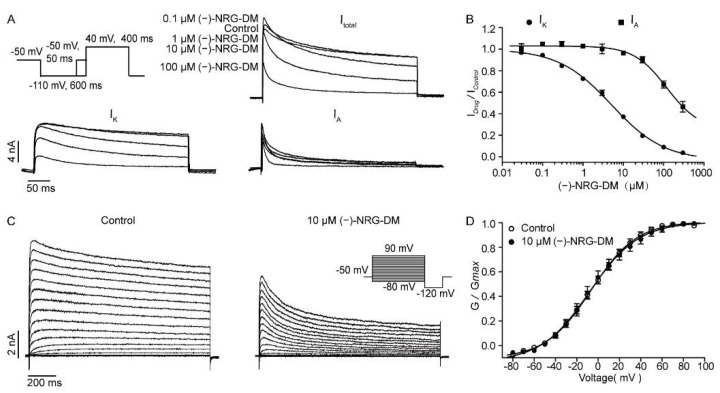
Inhibitory effects of (−)-NRG-DM on the potassium currents of DRG neurons in mice. (**A**) Typical traces of the transient outward potassium currents I_A_ and the delayed rectifier potassium currents I_K_ in the presence of (−)-NRG-DM at indicated concentrations. (**B**) The dose–response curve of (−)-NRG-DM on I_K_ and I_A_ currents. The IC_50_ values were 5.10 ± 0.04 μM and 119.90 ± 0.03 μM, respectively (*n* = 5). (**C**) Representative activation current traces of Kv channels before and after 10 μM (−)-NRG-DM. (**D**) Activation curves of Kv currents before and after 10 μM of (−)-NRG-DM (*n* = 6).

(−)-NRG-DM treatment caused a significant decrease in the current threshold for DRG neurons indicating that the compound may inhibit the potassium currents. The currents in mouse DRG neurons can be separated into I_A_ and I_K_. They could be distinguished by applying voltage steps from a holding potential of −50 mV, at which I_A_ was almost completely inactivated, while I_K_ remained unchanged. Thereby, I_A_ could be separated by subtracting I_K_ from the total potassium currents. According to these electrophysiological characteristics, the effects of (−)-NRG-DM on potassium currents were examined (Figure 5A). We found that (−)-NRG-DM inhibited potassium currents in a dose-dependent manner, and the IC_50_ values were 5.10 ± 0.04 μM and 119.90 ± 0.03 μM for I_K_ and I_A_, respectively (Figure 5B, *n* = 5). The data indicated that (−)-NRG-DM is an inhibitor of Kv channels in DRG neurons. Additionally, the effects of (−)-NRG-DM on the kinetics of the Kv channels were further characterized. The Kv currents were elicited by multiple 1500 ms depolarization pulses ranging from −80 mV to +90 mV in 10 mV increments from a holding potential of −80 mV. Congruently, the amplitudes of elicited potassium currents were potently reduced by (−)-NRG-DM at 10 µM, a concentration around IC_50_ of (−)-NRG-DM on I_K_ currents (Figure 5C). The activation curves of Kv channels before and after the perfusion of 10 µM (−)-NRG-DM were fitted with the Boltzmann equation, the data showed that (−)-NRG-DM does not affect the activation of the potassium currents (Figure 5D). The values of V_1/2_ in the absence and presence of 10 µM (−)-NRG-DM were −6.43 ± 1.21 mV and −8.08 ± 2.02 mV, respectively (Figure 5D). These data showed that (−)-NRG-DM is an inhibitor of native potassium currents in DRG neurons.

### 2.6. Inhibition of (−)-NRG-DM on Neuronal Sodium Currents

**Figure 6:**
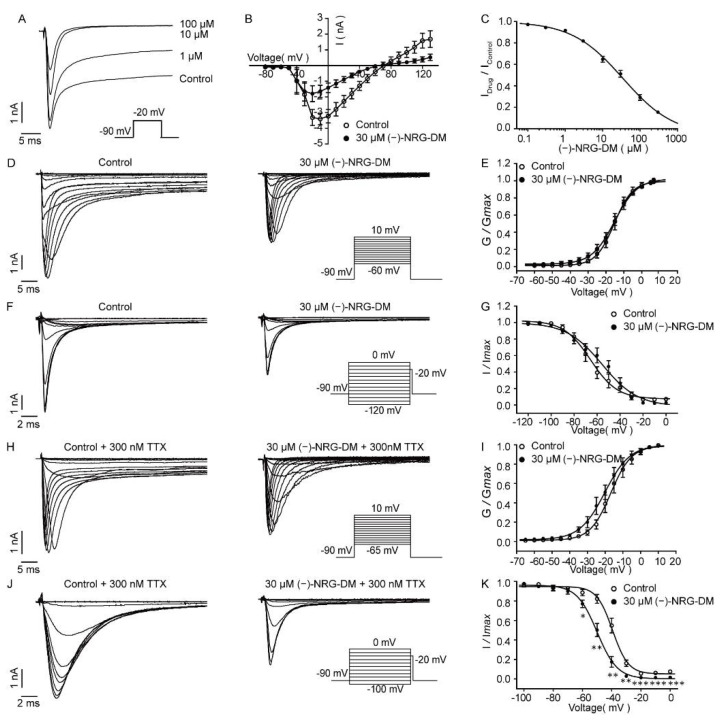
Characterization of (−)-NRG-DM inhibition on Nav currents of DRG neurons in mice. (**A**) Representative traces of Nav currents in the absence or presence of (−)-NRG-DM at indicated concentrations. (**B**) Current–voltage relationship of Nav currents with or without 30 μM (−)-NRG-DM (*n* = 8). (**C**) The dose–response curve of (−)-NRG-DM on native Nav currents. The IC_50_ value was 34.78 ± 0.14 μM (*n* = 5). Typical activation current traces (**D**) and steady-state activation curves (**E**) of total Nav currents before and after application of 30 µM (−)-NRG-DM (*n* = 6). Representative inactivation traces (**F**) and steady-state inactivation curves (**G**) of total Nav currents before and after perfusion of 30 μM (−)-NRG-DM (*n* = 6). Typical activation current traces (**H**) and steady-state activation curves (**J**) of TTX-R currents before and after perfusion of 30 µM (−)-NRG-DM (*n* = 7). Representative inactivation traces (**I**) and steady-state inactivation curves (**K**) of TTX-R currents before and after perfusion of 30 μM (−)-NRG-DM (*n* = 7). Statistical significance: * *p* ≤ 0.05, ** *p* ≤ 0.01, *** *p* ≤ 0.001.

A reduction in amplitudes of action potentials manifested that (−)-NRG-DM may inhibit native sodium currents, which are mainly involved in the rising phase of an action potential [39]. To record native sodium currents in acutely isolated DRG neurons, a 50 ms test pulse depolarized to −20 mV from a holding potential of −90 mV was applied. As illustrated in Figure 6A, the amplitudes of peak currents and persistent currents dose-dependently declined as the concentration of (−)-NRG-DM increased. The IC_50_ value of (−)-NRG-DM on native Nav currents was 34.78 ± 0.14 μM (Figure 6C, *n* = 5). To understand how (−)-NRG-DM inhibited Nav channels, the effects of 30 µM compound (−)-NRG-DM on Nav channel kinetics were further characterized. The activation currents were elicited by applying step pluses ranging from −60 mV to +15 mV in 5 mV increments for a 50 ms period from a holding potential of −90 mV (Figure 6D). The activation curves showed that the half-activation voltage did not change significantly after the perfusion of 30 μM (−)-NRG-DM, and the V_1/2_ values were −15.34 ± 0.58 mV and −15.23 ± 0.56 mV, respectively (Figure 6E). The influence of 30 µM (−)-NRG-DM on steady-state inactivation was assessed by 500 ms conditioning pulses ramping from −120 mV to 0 mV in 10 mV increments, followed by a 20 ms test pulse at −20 mV (Figure 6F). In contrast to the lack of effect on the Nav channel activation, (−)-NRG-DM caused a depolarization shift of the steady-state inactivation. The V_1/2_ value was shifted from −65.72 ± 1.74 mV to −55.34 ± 2.14 mV by 30 µM (−)-NRG-DM (Figure 6G). These data showed that (−)-NRG-DM is an inhibitor of native sodium channels in small-diameter DRG neurons.

The sodium currents in small-diameter DRG neurons could be furtherly subdivided into TTX-sensitive (TTX-S) and TTX-resistant (TTX-R) currents, which contribute to setting the firing threshold and the rising phase of an action potential [40]. To isolate TTX-R currents, whole-cell sodium currents were measured in the presence of 300 nM TTX. Similar to its effect on total sodium currents, (−)-NRG-DM did not affect the activation either (Figure 6I). The values of V_1/2_ in the absence and presence of 30 μM (−)-NRG-DM were −16.82 ± 0.72 mV and −20.14 ± 0.87 mV, respectively. Notably, (−)-NRG-DM caused a hyperpolarizing shift in steady-state inactivation. The value of V_1/2_ shifted from −39.43 ± 0.66 mV to −49.99 ± 0.67 mV by 30 µM (−)-NRG-DM (Figure 6K). These data indicated that (−)-NRG-DM is an inhibitor of sodium currents and preferentially affects channel inactivation. 

### 2.7. Inhibitory Effects of (−)-NRG-DM on Heterologously Expressed Kv2.1 Channel

**Figure 7:**
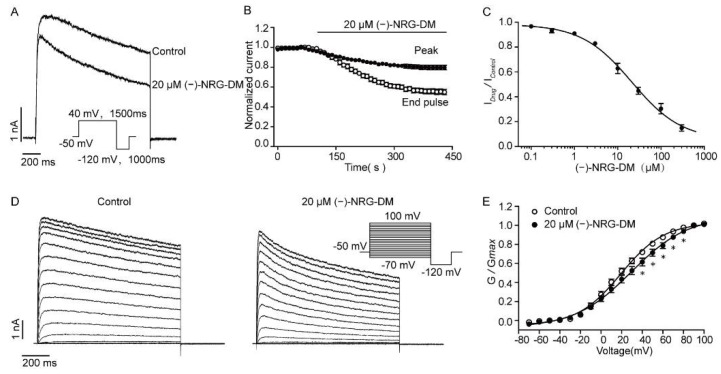
Inhibitory effects of (−)-NRG-DM on Kv2.1 channels heterologously expressed in CHO cells. (**A**) Representative Kv2.1 current traces in the absence and presence of 20 µM (−)-NRG-DM (Inset) The recording protocol. (**B**) Time course of peak and end-pulse currents of Kv2.1 channels before and after perfusion of 20 µM (−)-NRG-DM. (**C**) The dose–response curve of (−)-NRG-DM on Kv2.1 channel. The IC_50_ value was 21.17 ± 0.11 µM (*n* = 5). (**D**) Representative activation current traces of Kv2.1 channels in absence and presence of 20 µM (−)-NRG-DM (Inset) The recording protocol. (**E**) Activation curves of Kv2.1 channels with or without 20 µM (−)-NRG-DM (*n* = 5). Statistical significance: * p ≤ 0.05.

Kv2.1 represents a major component of I_K_ currents in neurons and plays an important role in the formation of functional Kv channels [18,41]. The potent inhibitory effects of (−)-NRG-DM on native potassium currents in DRG neurons prompted us to investigate whether the natural analgesic compound affects Kv2.1 channels transiently expressed in CHO cells. Congruently, (−)-NRG-DM dose-dependently inhibited Kv2.1 channels with an IC_50_ value of 21.17 ± 0.11 µM (Figure 7A, *n* = 5). The typical Kv2.1 current was elicited by applying a 40 mV depolarization stimulus before and after application of (−)-NRG-DM at 20 µM, a concentration around the IC_50_ of Kv2.1 channels, as illustrated in Figure 7B. The effects of 20 µM (−)-NRG-DM on the activation of Kv2.1 channels were furtherly evaluated. The activation currents of Kv2.1 were elicited by applying multiple pulses ranging from −80 mV to +110 mV in 10 mV increments for a 1500 ms period from a holding potential of -50 mV (Figure 7D). Surprisingly, the value of V_1/2_ shifted from 22.30 ± 1.12 mV in the control condition to 35.88 ± 1.88 mV in the presence of 20 µM (−)-NRG-DM (Figure 7E). These data showed that (−)-NRG-DM is an inhibitor of the Kv2.1 channel and dampens channel activation.

### 2.8. (−)-NRG-DM Inhibits Nav Channels

**Figure 8:**
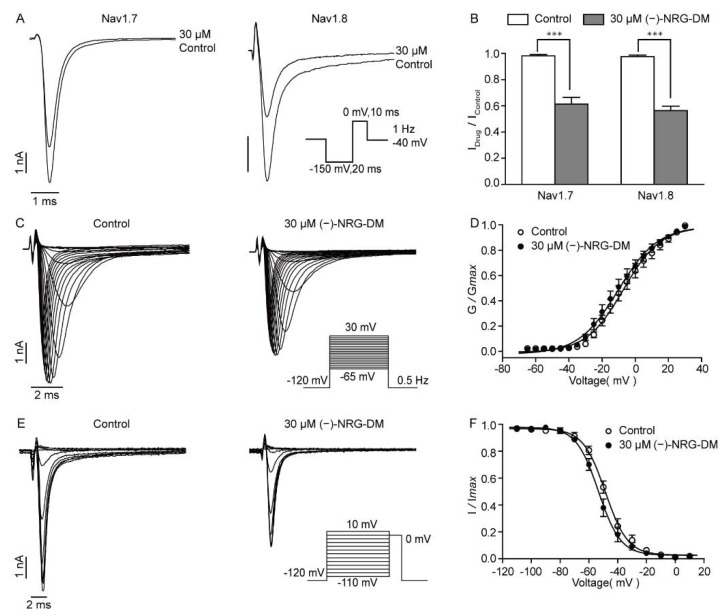
Inhibitory effect of (−)-NRG-DM on Nav currents. (**A**) Representative Nav1.7 and Nav1.8 current traces in the absence and presence of 30 µM (−)-NRG-DM recorded with the depicted protocol. (**B**) Summarized data showing the suppression effect of (−)-NRG-DM (30 µM) on Nav1.7 and Nav1.8 channels (*n* = 5). (**C**) Representative Nav1.8 current traces in absence and presence of 30 µM (−)-NRG-DM recorded with the protocol shown inset. (**D**) Activation curves obtained in the absence and presence of 30 µM (−)-NRG-DM (*n* = 13). (**E**) Representative activation current traces of Nav1.8 currents before and after 30 μM (−)-NRG-DM. (**F**) Steady-state inactivation curve of Nav1.8 currents before and after 30 μM (−)-NRG-DM (*n* = 13). Statistical significance: *** *p* ≤ 0.001.

The dose-dependent suppression of the sodium currents by (−)-NRG-DM indicated that it should inhibit the Nav channels. As Nav1.7 and Nav1.8 are mainly distributed in the PNS and have been demonstrated to play an important role in pain sensing, we detected the effects of (−)-NRG-DM on Nav1.7 and Nav1.8 channels stably expressed in HEK293 cells. As shown in Figure 8A, we found that 30 μM (−)-NRG-DM, a concentration around the IC_50_ of native Nav currents in DRG neurons, can potently inhibit Nav1.7 and Nav1.8 currents. The values of I_Drug_/I_Control_ were 0.61 ± 0.05 and 0.56 ± 0.03, respectively (Figure 8B). There is no significant difference in the inhibitory efficacy between Nav1.7 and Nav1.8 channels; the data showed that (−)-NRG-DM is a non-selective Nav channel inhibitor (Figure 8B). To understand how (−)-NRG-DM inhibits Nav1.8 channels, we assessed its impacts on the voltage dependence of steady-state activation and inactivation. The Nav1.8 currents were elicited by applying step pluses ranging from −65 mV to +30 mV for 200 ms in 5 mV increments at a stimulus frequency of 0.5 Hz (Figure 8C). Activation curves showed that the V_1/2_ did not change significantly before and after the perfusion of 30 µM (−)-NRG-DM, which was −7.10 ± 1.10 mV and −10.31 ± 1.17 mV, respectively (Figure 8D). The influence of (−)-NRG-DM on steady-state inactivation was evaluated by applying a 500 ms conditioning pulse ramping from −110 mV to 10 mV in 10 mV increments, followed by a 20 ms test pulse at −20 mV (Figure 8E). The values of V_1/2_ were −48.58 ± 0.67 mV and −53.37 ± 0.70 mV before and after the application of 30 µM (−)-NRG-DM (Figure 8F). Similarly, no significant difference was observed. These data showed that (−)-NRG-DM is a nonselective inhibitor of Nav channels.

## 3. Discussion

In the present study, a naringenin derivative named (−)-NRG-DM was first isolated from *N. jatamansi* (Figure 1). Intraperitoneal administration of (−)-NRG-DM dose-dependently attenuated pain in a formalin-induced mouse inflammatory pain model and mustard oil-induced mouse colorectal pain model, which, respectively, corresponded to somatic pain and visceral pain (Figure 2 and Figure 3). Notably, (−)-NRG-DM was well tolerated as no significant neurotoxicity was observed at doses of 100 mg/kg, 200 mg/kg, and even 400 mg/kg in the rotarod test (Table 1). All animals displayed no sign of toxicity during the rotarod test (0.5 and 1 h) and 24 h after the drug administration. The data showed that (−)-NRG-DM is an analgesic compound with a wide margin of safety. Combining the data obtained from the acutely isolated mouse small-diameter DRG neurons, heterologous expression system, and pharmacokinetic study, we furtherly elucidated that inhibition of neuronal channels mediating I_K_ and I_Na_ currents is implicated in the (−)-NRG-DM-produced analgesic activity.

The current study showed that the analgesic (−)-NRG-DM directly dampens neuron excitability in acutely isolated mouse small-diameter DRG neurons with a reduced threshold and amplitude of action potential firing (Figure 4A). Intriguingly, the suppression of firing frequency is tightly accompanied by a depolarized firing threshold (Figure 4A,D). The significant reduction in the number of action potentials started after 10 µM (−)-NRG-DM was applied, at which the significant difference in the firing threshold occurred (Figure 4D,E). The data suggested that (−)-NRG-DM may cause suppression through a mechanism similar to neuronal desensitization. The feature is involved in capsaicin, a TRPV1 channel agonist, and an 8% capsaicin patch (Qutenza) has been approved for the treatment of chronic pain in clinics [42]. Analgesic activities produced by a direct desensitization of nociceptive neurons might reduce side effects including gastrointestinal erosions, renal and hepatic insufficiency which are commonly associated with cyclooxygenase inhibitors [43]. As 10 μM (−)-NRG-DM did not affect TRPA1 currents, the neuronal desensitization could not be ascribed to its modulation on the channels (Figure S1). The suppression of neuron activity produced by (−)-NRG-DM is in agreement with the study of (+)-naringenin 4′,7-dimethyl ether ((+)-NRG-DM), a naturally occurring naringenin derivative that has been isolated many times from plants, that showed analgesic activity in vivo and that did not influence the production and release of pro-inflammatory factors compared to other naringenin derivatives [44]. Additionally, radioligand binding assay demonstrated that (+)-NRG-DM does not show affinity to endocannabinoid or opioid receptors [22]. Together, our data showed that (−)-NRG-DM causes a direct inhibition of neuron excitability through a mechanism similar to excitatory desensitization in nociceptive neurons.

Voltage-gated potassium currents play a fundamental role in the modulation of resting membrane potential [45]. In the small-diameter DRG neurons, the currents can be divided into two separate components: I_A_ and I_K_ [46]. Due to the low current density and distribution ratio, the I_A_ currents did not seem to play a key role in the excitability of nociceptive neurons. The I_K_ currents contribute to the setting of resting membrane potential and appear to be the main contributor to after-hyperpolarization [20,47]. Consistent with the depolarized threshold of action potential firing, (−)-NRG-DM potently inhibited the I_K_ but not I_A_ currents with IC_50_ values of 5.10 ± 0.04 μM and 119.90 ± 0.03 μM, respectively (Figure 5A,B). Notably, the pharmacokinetic study showed that the plasma concentration of (−)-NRG-DM after 0.5~1.5 h intraperitoneal injection of the compound at 50 mg/kg is around 7 µM, which is higher than the IC_50_ value of (−)-NRG-DM on I_K_ currents (Table 2). The reduction in the I_K_ currents in DRG neurons at this concentration was around 58% (Figure 5B). Kv2.1 homotetramers or containing heterotetramers represent the major component of I_K_ currents in neurons [18,40]. Consistently, the IC_50_ value of (−)-NRG-DM on Kv2.1 channels heterologously expressed in CHO cells was 21.17 ± 0.11 µM (Figure 7A,B). The difference between the IC_50_ values obtained from DRG neurons and CHO cells may emerge from species differences in primary pharmacology. One might argue that a reduction in the I_K_ currents has been found in multiple animal models for studying pain, and lesser I_K_ currents are involved in human labor pain [48,49]. The feature might be similar to that found in Nav1.9 channels, in which the gain-of-function mutations caused a loss of pain reception in humans and exhibited a reduced neuronal excitability during the long stimulus due to neuronal desensitization [50]. Thus, our data showed that the inhibition of I_K_ mainly contributes to (−)-NRG-DM-produced pain relief and Kv2.1-containing channels involved in the inhibitory activity.

The reduction in the amplitudes of the action potentials after exposure to (−)-NRG-DM was in accordance with its suppression of sodium currents (Figure 4C). (−)-NRG-DM inhibited Nav currents in DRG neurons in a dose-dependent manner with an IC_50_ value of 34.78 ± 0.14 µM (Figure 6C). Nevertheless, a reduction in the I_Na_ of the DRG neurons was around 28% at the plasma concentration obtained from the pharmacokinetic study (Figure 6C). Nav1.7 and Nav1.8 are analgesic-related Nav subtypes in the PNS, which separately correspond to the TTX-S and TTX-R currents in small-diameter DRG neurons [46]. The inhibitory ratios of (−)-NRG-DM (30 µM) on Nav1.7 and Nav1.8 were 39 ± 5% and 44 ± 3% respectively, which were very close to those on native sodium currents (Figure 8A,B). As an enantiomer, NRG-DM extracted from peanut stem and leaf promoted sleep by dampening neuronal excitability. In cortical neurons, the inhibitory activity of NRG-DM on Nav channels appeared to be more potent than that on Kv channels [51]. However, in the current study, due to the higher IC_50_ value, our data showed that inhibition of Nav channels might contribute to (−)-NRG-DM-produced analgesic activity but the effect is secondary to that of Kv channels.

In conclusion, our study showed that (−)-NRG-DM is a promising analgesic drug candidate potently attenuating somatic and visceral pain in vivo. The analgesic activity could be ascribed to its direct suppression of nociceptive neuron excitability through a desensitization mechanism. Due to the plasma concentration of (−)-NRG-DM being higher than the effective concentrations for the I_K_ and I_Na_, our study suggested that the inhibition of neuronal channels mediating these currents contributes to the analgesic activity. (−)-NRG-DM may act as a new structural framework for the subsequent development of analgesic drugs.

## 4. Materials and Methods

### 4.1. Chemical Compounds

(−)-NRG-DM, isolated from the roots of *N. jatamansi*, was prepared and certificated by Professor Jun-Li Yang’s lab at Lanzhou Institute of Chemical Physics, Chinese Academy of Sciences. The procedure of extraction and isolation of (−)-NRG-DM can be found in the Supporting Information. The compound was dissolved and stored in dimethyl sulfoxide (DMSO) to produce a 20 mM stock solution and then diluted in bath solution to obtain final concentrations. DMSO, at the final concentrations (≤0.5%), was well tolerated with no observable toxic effects on cells and neurons. To conduct research on animals, (−)-NRG-DM was dissolved in a mixture of 5% DMSO, 5% Tween 80, and 90% (0.9% NaCl). TTX was purchased from Qinhuangdao Aquaculture Technical Developing Company (Qinhuangdao, China). All other chemicals were purchased from Sigma-Aldrich (St Louis, MO, USA).

### 4.2. Animals

All mice were obtained from the Beijing Vitalriver Laboratory Animal Technology Co., Ltd. (Beijing, China). Mice were housed and assayed under controlled temperature conditions (22 ± 2 °C) and a 12 h light/dark cycle with free access to food and water. All animal procedures were performed according to the National Institutes of Health Guide for the Care and Use of Laboratory Animals and were strictly followed and approved by the guidelines of the IACUC (Institutional Animal Care and Use Committees). The IACUC checked all protocols and approved this study. The animal experiments were conducted in a blinded manner, i.e., drug administration and behavioral tests were finished by different investigators.

### 4.3. Formalin-Induced Inflammatory Model

Adult male ICR mice weighing 20 ± 2 g were randomly divided into 3 groups (*n* = 10 for each group) and acclimatized in a transparent observation chamber for at least 30 min before the experiment. Mice were intraperitoneally administrated with vehicle or (−)-NRG-DM (30 or 50 mg/kg) 30 min prior to formalin injection. Then, 1% formalin solution (20 μL per site) was subcutaneously injected into the plantar of the left hind paw to induce acute inflammatory pain. Immediately, mice were put back into the observation chambers. Nociception caused by formalin was assessed by scoring painful behaviors and licking time over a period of 60 min. In the present study, the score represented the sum of weighted formalin-induced pain-related behavior: 1 = flinching, 2 = shaking, and 3 = licking or biting of the injected paw. Phases were defined as follows: the peak time of the early nociceptive response phase (phase I) was 0~10 min, and the late phase (phase II) was 11~60 min after formalin injection.

### 4.4. Mustard Oil-Induced Mouse Colorectal Pain Model

Male C57BL/6 mice, weighing 23~25 g, were randomly assigned into 3 groups (*n* = 10 for each group) and placed in a transparent observation chamber for at least 20 min prior to the experiment. Mice were intraperitoneally administrated with vehicle or (−)-NRG-DM (30 or 50 mg/kg) 30 min before the injection of diluted mustard. Subsequently, 50 µL of diluted mustard oil solution (0.75% in 70% ethanol) was intracolonically administrated and the number of pain responses was counted for 30 min. In the present study, postures defined as pain-related behaviors were in agreement with previous descriptions: (1) licking of the abdomen, (2) stretching the abdomen, (3) squashing of lower abdomen against the floor, (4) abdominal wall retractions.

### 4.5. Rotarod Test

To determine the neurotoxicity effects of (−)-NRG-DM, the standardized rotarod test was conducted in male ICR mice weighted 20 ± 2 g. The mice were divided at random into 4 groups (*n* = 10 for each group). The mice were placed on a rotarod appliance (YLS-4C, Bio-will, Shanghai, China) with a rod of 3 cm diameter, rotating at a constant speed of 6 rpm. The day before the compound test, all mice were pre-trained and only the animals able to remain on the rod for at least 1 min every time in three consecutive trials (3 min) were retained. During the test, the mice were measured in the rotarod test 0.5 h and 1 h after intraperitoneal administration of (−)-NRG-DM. The animals unable to remain on the rod for 3 consecutive periods were considered motor coordination impaired.

### 4.6. Pharmacokinetic Study

The pharmacokinetic study was performed in male ICR mice weighted 20 ± 2 g. The mice were fasted for 12 h before intraperitoneal administration of (−)-NRG-DM at 50 mg/kg. Blood samples (0.5 mL) were collected at 0.5, 1, and 1.5 h from the abdominal aorta into the heparinized tubes after drug administration. The plasma was separated by centrifugation (11,000 rpm for 5 min) and then stored at −80 °C until analyzed. Plasma samples (20 μL) were treated with the addition of the internal standard solution (20 μL) and acetonitrile (300 μL), then vortexed for 15 min (1000 rpm, RT), and centrifuged of 15 min (3700 rpm, 4 °C). The supernatants were collected for analysis using LC−MS/MS. The data were processed using Analyst software version 1.6.3 (Sciex, ON, Canada).

### 4.7. Preparation of Dorsal Root Ganglion Neurons

Dorsal root ganglia (DRG) were dissected from male C57BL/6 mice aged 4~6 weeks. The ganglia were first cut into small pieces and then digested at 37 °C for 20 min in DMEM containing 1 mg/mL collagenase type I and 0.25 mg/mL trypsin (Sigma-Aldrich). Subsequently, the digested small fragments were terminated and resuspended with DMEM/F12 growth medium (Gibco) supplemented with 10% fetal bovine serum (FBS) (Gibco). Finally, the dissociated DRG neurons were seeded onto 24-well plates with poly-L-lysine-coated coverslips and placed in a 37 °C, 5% CO_2_ incubator for at least 1 h before electrophysiological experiments.

### 4.8. Cell Culture and Transfection

Human embryonic kidney 293 (HEK293) cells stably expressing human Nav1.7 and Nav1.8 channels were grown in a high-glucose DMEM medium (Gibco) containing 10% FBS (Gibco). The media were respectively supplemented with 50 mg/mL and 100 mg/mL hygromycin B (Invitrogen, Carlsbad, CA, USA). The cDNA encoding human Kv2.1 channels was synthesized by Sangon Biotech Co., Ltd. (Shanghai, China) based on the GenBank (Kv2.1 Gene ID: 25736) and was subcloned into the pcDNA3.1(+) vector. Chinese hamster ovary (CHO) cells were cultured in DMEM/F12 medium (Gibco) supplemented with 10% FBS. To transiently express the Kv2.1 channels for electrophysiological studies, the constructs encoding the EGFP and the Kv2.1 were co-transfected into the CHO cells with Lipofectamine 2000 reagent (Invitrogen) referring to the manufacturer’s instructions. The transfected cells were seeded onto poly-L-lysine-coated glass coverslips before they were used for the electrophysiological study. All cells were grown under standard tissue culture conditions (5% CO_2_, 37 °C).

### 4.9. Electrophysiological Recordings

A standard whole-cell voltage-clamp technique was used to record membrane currents from the heterologous expression cells and the acutely isolated DRG neurons. A standard whole-cell current-clamp mode was applied to record the action potential (AP) firing in DRG neurons. Pipettes were pulled from borosilicate glass capillaries and the resistances of pipettes were 3~5 MΩ when they were filled with the intracellular solution and placed in the bath. For the recording of potassium currents from the transfected CHO cells, the pipette or intracellular solution contained (in mM): 145 KCl, 10 HEPES, 1 MgCl_2_, 5 EGTA, 1 CaCl_2_, and 10 HEPES (pH 7.2 adjusted by KOH); bath or extracellular solution contained (in mM): 140 NaCl, 5 KCl, 2 CaCl_2_, 1 MgCl_2_, 10 glucose, and 10 HEPES (pH 7.4 adjusted by NaOH). For the recording of sodium currents from the stable cell lines, the pipette or intracellular solution contained (in mM): 140 CsF, 10 NaCl, 20 glucose, 1.1 EGTA, and 10 HEPES (pH 7.3 adjusted with CsOH); bath or extracellular solution contained (in mM): 140 NaCl, 3 KCl, 1 CaCl_2_, 1 MgCl_2_·6H_2_O, 20 glucose, and 10 HEPES (pH 7.3 adjusted by NaOH). For recordings of the action potential firing properties and potassium currents in DRG neurons, the pipette or intracellular solution contained (in mM): 140 KCl, 1 CaCl_2_, 1 MgCl_2_, 10 EGTA, and 10 HEPES (pH 7.2 adjusted with KOH); the extracellular solution contained (in mM): 140 NaCl, 5 KCl, 1 CaCl_2_, 1.25 MgCl_2_, 10 glucose, and 10 HEPES (pH 7.4 adjusted with NaOH). For the recording of neuronal sodium currents from DRG neurons, the pipette or intracellular solution contained (in mM): 120 CsCl, 10 NaCl, 10 TEA-Cl, 1 CaCl_2_, 1 MgCl_2_, 10 EGTA, and 10 HEPES (pH 7.2 adjusted with CsOH); bath or extracellular solution contained (in mM): 120 NaCl, 5 KCl, 1 CaCl_2_, 1.25 MgCl_2_, 20 TEA-Cl, 10 glucose, and 10 HEPES (pH 7.4 adjusted by NaOH). During the recordings, a BPS perfusion system (ALA Scientific Instruments, Westbury, NY, USA) was used to continuously perfuse bath solutions. Data acquisition was performed at room temperature with the Axopatch-200B amplifier (Axon Instruments, Burlingame, CA, USA), and the signals were filtered at 2 kHz and digitized with a Digidata 1440 A interface (Axon Instruments) at 50 kHz.

### 4.10. Statistics

Patch-clamp data were processed using Clampfit 10.6 (Molecular Device, Sunnyvale, CA, USA) and then analyzed in GraphPad Prism 5 (GraphPad Software, San Diego, CA, USA). Voltage-dependent activation curves were fitted to the Boltzmann equation: G = G_min_ + (G_max_ − G_min_)/[1 + exp (V − V_1/2_)/S], where G_max_ is the maximum conductance, G_min_ is the minimum conductance, V_1/2_ is the voltage to reach 50% of the maximum conductance, and S is the slope factor. Steady-state inactivation curves were constructed by plotting the normalized peak currents during the test pulses as a function of the prepulse potentials. The data were fitted to the Boltzmann equation: I/I_max_ = 1/{1+ exp [(V − V_1/2_)/K*i*]}, where I is the amplitude of peak currents at each voltage, I_max_ is the maximal value of peak currents, V and V_1/2_ are the prepulse potential and the half-maximal potential for inactivation, respectively, and K*i* is the inactivation slope factor. Dose–response curves were fitted with the Hill equation: Y = Bottom + (Top − Bottom)/{1+10[(LogIC_50_ − X) × k]}, where Bottom and Top are the minimum and maximum inhibition, respectively; X is the log of the concentration; Y is the value of I_Drug_/I_Control_; IC_50_ is the drug concentration producing a half-maximum response, k is the Hill Slope. The data are shown as the mean ± SEM, and the significance was estimated using paired two-tailed Student’s *t*-test unless otherwise stated. Statistical significance: * *p* ≤ 0.05, ** *p* ≤ 0.01, *** *p* ≤ 0.001.

## Figures and Tables

**Table 1:** Effects of (−)-NRG-DM on motor impairment in the rotarod test in ICR mice.

Compound	Dosage (mg/kg)	Time of Test (h)	Fall n_Fall_ /n_Test_	Motor Impairment (%)
Vehicle	-	0.5	0/10	0
		1	0/10	0
(−)-NRG-DM	100	0.5	0/10	0
		1	0/10	0
	200	0.5	1/10	10
		1	0/10	0
	400	0.5	0/10	0
		1	0/10	0

**Table 2:** Mean plasma concentration of (−)-NRG-DM administrated via intraperitoneal route in ICR mice.

Compound	Dosage (mg/kg)	Time of Test (h)	Concentration (ng/mL)	Concentration (μM/L)
		0.5	2293.3 ± 183.4	7.64 ± 0.61
(−)-NRG-DM	50	1	2216.0 ± 1252.0	7.39 ± 4.17
		1.5	716.7 ± 222.5	2.39 ± 0.74

Data are presented as the mean ± SEM.

## Data Availability

The data presented in this study are available upon request from the corresponding author.

## Supplementary Materials

The following supporting information is available online, Figure S1: The effects of (−)-NRG-DM on TRPA1 channels; Figure S2: HRESIMS spectrum of (−)-NRG-DM; Figure S3: ^1^ H NMR spectrum of (−)-NRG-DM (400 MHz, CDCl_3_); Figure S4: ^13^ C NMR spectrum of (−)-NRG-DM (100 MHz, CDCl_3_).

## Sample Availability

Samples of the compounds prepared in this article are available from the authors.
