# Supplementary material for: (−)-Naringenin 4′,7-dimethyl Ether Isolated from Nardostachys jatamansi Relieves Pain through Inhibition of Multiple Channels
Source: Molecules. 2022 Mar 7;27(5):1735. doi: 10.3390/molecules27051735 (PMC8911579; doi:10.3390/molecules27051735)
Supplement: Supplementary file 1 [file molecules-27-01735-s001.zip › molecules-1595875-supplementary.pdf]

Supplementary Materials

1. Experimental Section

Cell culture, Electrophysiology and Data analysis

HEK293 cells stably expressing human TRPA1 channels were cultured in a high-glucose DMEM medium (Gibco) containing 10% FBS (Gibco) and were selected 400 μg/mL of the antibiotic Hygromycin B (Invitrogen) and 15 μg/mL Blasticidin under standard tissue culture conditions (5% CO_2, 37 °C). The cell line was induced with 1 μg/mL doxycycline (Invitrogen) 24 h prior to the experiment. A standard whole-cell voltage-clamp technique was used to record TRPA1 currents from the cell lines. Pipettes were pulled from borosilicate glass capillaries and the resistances of pipettes were 3 ~ 5 MΩ when they were filled with the intracellular solution and placed in the bath. The pipette or intracellular solution contained (in mM): 140 CsCl, 10 HEPES, 5 EGTA, 0.1$CaCl_2$ and 1 $MgCl_2$ (pH 7.2 adjusted by CsOH); bath or extracellular solution contained (in mM): 140 NaCl, 5 KCl, 0.5 EGTA, 1 $MgCl_2$, 10 Glucose and 10 HEPES (pH 7.4 adjusted by NaOH). TRPA1 currents development was monitored with repetitive injections of 300 ms duration voltage ramps from − 100 to +100 mV every 2 s and the holding potential was set to 0 mV for 50 ms, existing in both sides of the voltage ramp. Data acquisition was performed at room temperature using EPC-10 amplifier and Patch Master Software (HEKA), and the signals were sampled at 10 kHz and low-pass filtered at 2.9 kHz. Compounds including AITC (allyl isothiocyanate), HC030031, or (−)-NRG-DM were dissolved in the bath solution and applied using a multi-barrel solution exchanger. Data were presented as the mean ± SEM. Statistical analyses were performed using Student's *t*-test (GraphPad Prism 5 Software). Asterisks (*) indicate statistically significant differences from the control group (*$P < 0.05$, **$P < 0.01$ and ***$P < 0.001$).

General Experimental Procedures

Optical rotations were recorded on an IP-digi300/2 polarimeter (InsMark, Shanghai, China). The nuclear magnetic resonance (NMR) spectra were recorded at 25 °C at 400 MHz for ^{1}H and 100 MHz for ^{13}C on a Bruker Avance III-400 spectrometer and the Bruker topspin 2.1 software was used to process the NMR spectra. High-resolution electrospray ionization mass spectrometry (HR-ESIMS) was obtained on a Bruker microTOF-Q II mass spectrometer. The silica gel of 200–300 mesh was purchased from Qingdao Marine Chemical Factory of China. Prep-HPLC separation was performed on a prep-HPLC

made by Hanbon Sci & Tech of China. TLC detections were conducted by spraying with 5% H_2SO_4 in ethanol (v/v) and then heating.

Plant Material

The roots and rhizomes of *Nardostachys jatamansi* DC. were purchased from the Yellow River medicinal materials market of Lanzhou in 2017. The sample was botanically identified by Prof. Huan-Yang Qi and a voucher specimen (NCB-HHTS-201701) has been deposited at the CAS Key Laboratory of Chemistry of Northwestern Plant Resources.

Extraction and Isolation

The dried roots and rhizomes of *N. jatamansi* (10 kg) were powered and extracted with methanol at room temperature (3 × 100 L, each for 7 days), and the extract was evaporated to dryness to obtain a 1.8 kg extract. Then, the extract was suspended in water and partitioned with petrol ether (PE, 3 × 1.0 L), EtOAc (3 × 1.0 L), and *n*-BuOH (3 × 1.0 L), respectively. The EtOAc part (220.0 g) was separated by silica gel column chromatography with a gradient of PE/ EtOAc (10:0-0:10) to yield nine fractions (Fr. 1-Fr. 9). Colorless columnar crystals were precipitated in the third fraction. After removing the crystals with acetone, the remaining part of Fr. 3 (6.2 g) was separated by silica gel CC and eluted with a gradient of PE/ EtOAc (10:1-1:1) to obtain twelve fractions (Fr. 3.1–Fr. 3.12). Fr. 3.2 (1.4 g) was further purified by HPLC (eluting with 72% MeOH in water, flow rate of 4 mL/min) to yield (−)-NRG-DM (980.0 mg, t_R = 19.7 min).

(−)-NRG-DM White needle crystal. $[\alpha]_D^{20}$ +7.8 (*c* 0.22 in CH_2Cl_2). ^1H NMR (400 MHz，$CDCl_3$) δ_H: 12.03 (1H, s, 5-OH), 7.38 (2H, d, *J* = 8.4 Hz, H-2', 6'), 6.95 (2H, d, *J* = 8.4 Hz, H-3', 5'), 6.06 (1H, s, H-8), 6.04 (1H, s, H-6), 5.37 (1H, dd, *J* = 12.8, 2.4 Hz, H-2), 3.83 (3H, s, OCH_3), 3.80 (3H, s, OCH_3), 3.10 (1H, dd, *J* = 17.2, 13.2 Hz, H-3a), 2.79 (1H, dd, *J* = 17.2，2.8 Hz，H-3b). ^{13}C NMR (150 MHz, $CDCl_3$) δ_C: 196.1 (C-4), 168.0 (C-7), 164.2 (C-5), 162.9 (C-9), 160.1 (C-4'), 130.4 (C-1'), 127.8 (C-2', 6'), 114.2 (C-3', 5'), 103.2 (C-10), 95.1 (C-6), 94.2 (C-8), 79.0 (C-2), 55.7, 55.4 (2 × OCH_3), 43.2 (C-3). (+)-HRESIMS: *m/z* 323.088 5 $[M + Na]^+$ (calcd. for $C_{17}H_{16}O_5Na$, 323.089 0), and 623.187 9 $[2M + Na]^+$ (calcd. for $C_{34}H_{32}O_{10}Na$, 623.188 8) [1-3].

2. References

1. Ya-yun Wang, Guo-xu Ma, Zhen Huang, Xiao-ming Zhong, Xu-dong Xu, Jing-quan Yuan. Identification of Compounds in Alien Invasive Plant Chromolaena odorata. *Chin Pharm J*. **2016**, 51(9): 698-702.
2. Kin-ichi Oyama, Tadao Kondo. Total synthesis of flavocommelin, a component of the blue supramolecular pigment from Commelina communis, on the basis of direct 6-C-glycosylation of flavan. *J. Org. Chem*. **2004**, 69, 5240-5246.
3. R. A. Zainullin, R. V. Kunakova, V. F. Gareev, I. V. Galyautdinov, Z. R. Sadretdinova, Z. S. Muslimov, V. N. Odinokov. Flavanones and flavones from Bashkir propolis. *Chem. Nat. Compd*. **2018**, 54(5): 975-977.

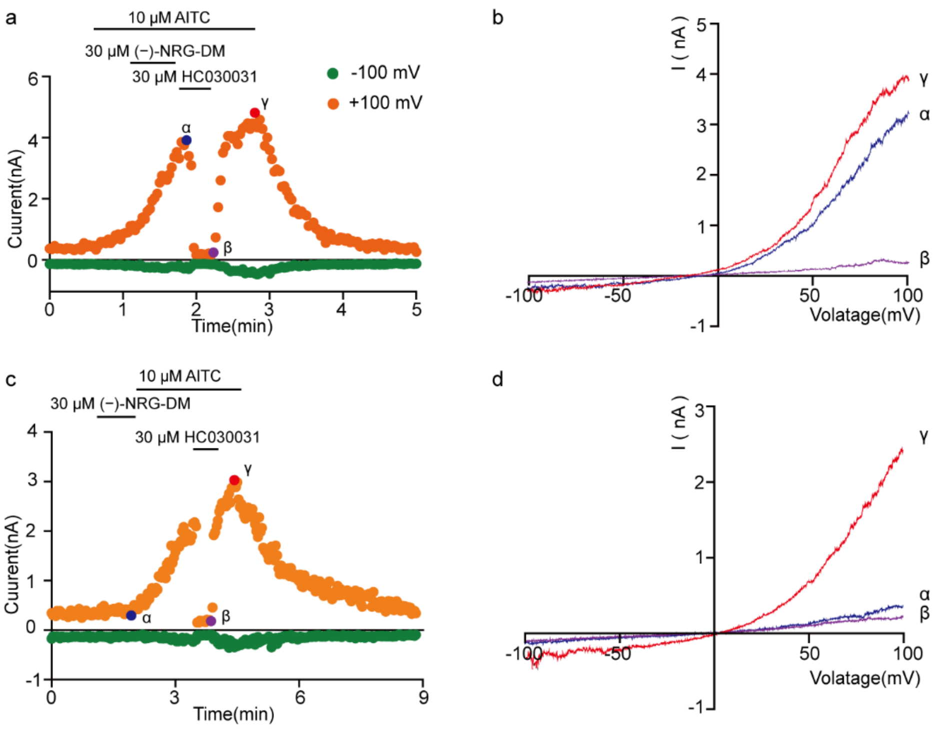

Figure S1. (−)-NRG-DM did not affect the TRPA1 channels. The HEK293 cells stably expressing human TRPA1 channels were used, the TRPA1 currents could be induced by 10 μM AITC (allyl isothiocyanate) and inhibited by 30 μM HC030031, which were used as TPRA1 channel activator and inhibitor, respectively. (−)-NRG-DM (30 μM) did not enhance any TRPA1 currents or inhibit AITC-induced TRPA1 currents either. Representative time course (a, c) and current traces and the current-voltage (I-V) relationships (b, d) of human TRPA1 currents in the presence of compounds at indicated concentration.

Mass Spectrum SmartFormula Report

Analysis Info

Analysis Name	D:\Data\user\mengxianhua 20211230-2.d	Acquisition Date	2021-12-30 17:20:50
Method	tune_low.m	Operator	BDAL@DE
Sample Name	gsg9	Instrument / Ser#	micrOTOF-Q 20453
Comment			

Acquisition Parameter

Source Type	ESI	Ion Polarity	Positive	Set Nebulizer	0.4 Bar
Focus	Active	Set Capillary	4500 V	Set Dry Heater	180 ¡ãC
Scan Begin	100 m/z	Set End Plate Offset	-500 V	Set Dry Gas	4.0 l/min
Scan End	2000 m/z	Set Collision Cell RF	300.0 Vpp	Set Divert Valve	Waste

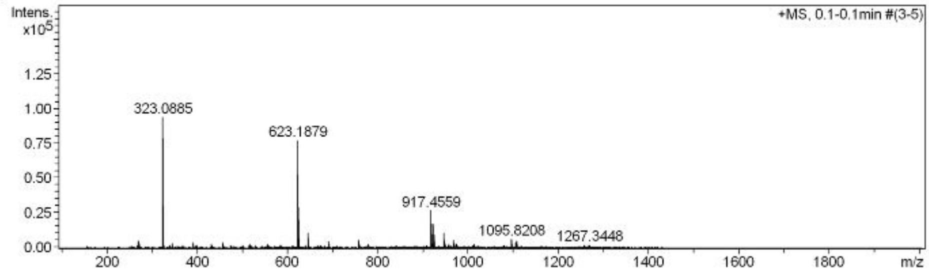

Meas. m/z	#	Formula	m/z	err [ppm]	Mean err [ppm]	rdb	N-Rule	e¡¥ Conf	mSigma	Std I	Std Mean m/z	Std I Var Norm	Std m/z Diff	Std Comb Dev
323.0885	1	C 17 H 16 Na O 5	323.0890	1.5	1.6	9.5	ok	even	8.8	15.8	0.6	6.5	0.1	842.7
623.1879	1	C 34 H 32 Na O 10	623.1888	1.5	2.2	18.5	ok	even	3.5	4.6	1.8	1.9	2.5	842.7

Figure S2. HRESIMS spectrum of (−)-NRG-DM

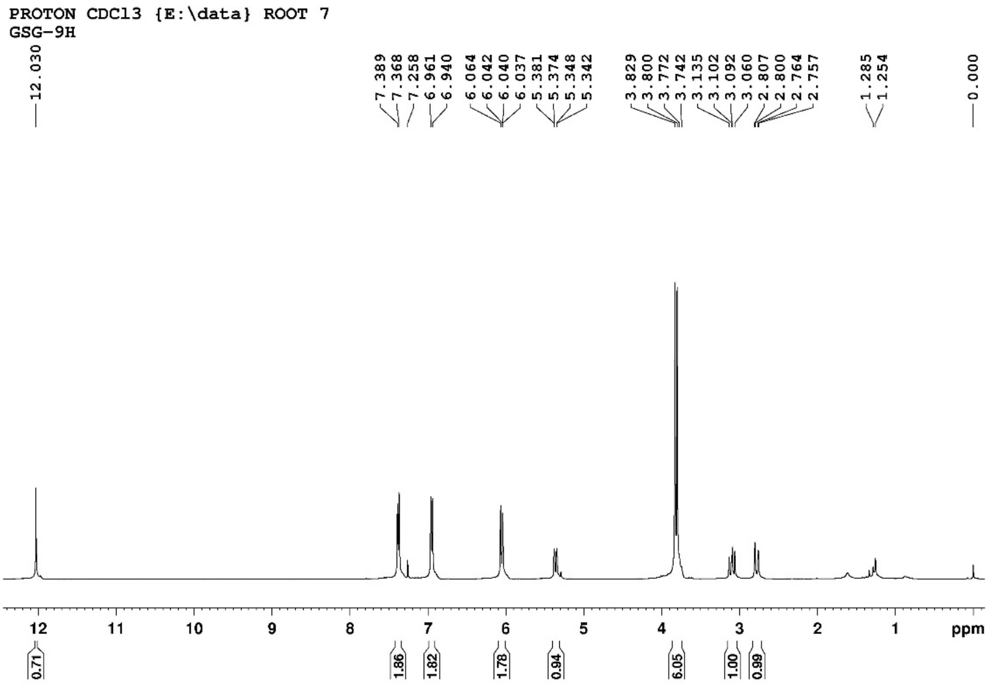

Figure S3. ^{1}H NMR spectrum of (−)-NRG-DM (400 MHz, $CDCl_3$)

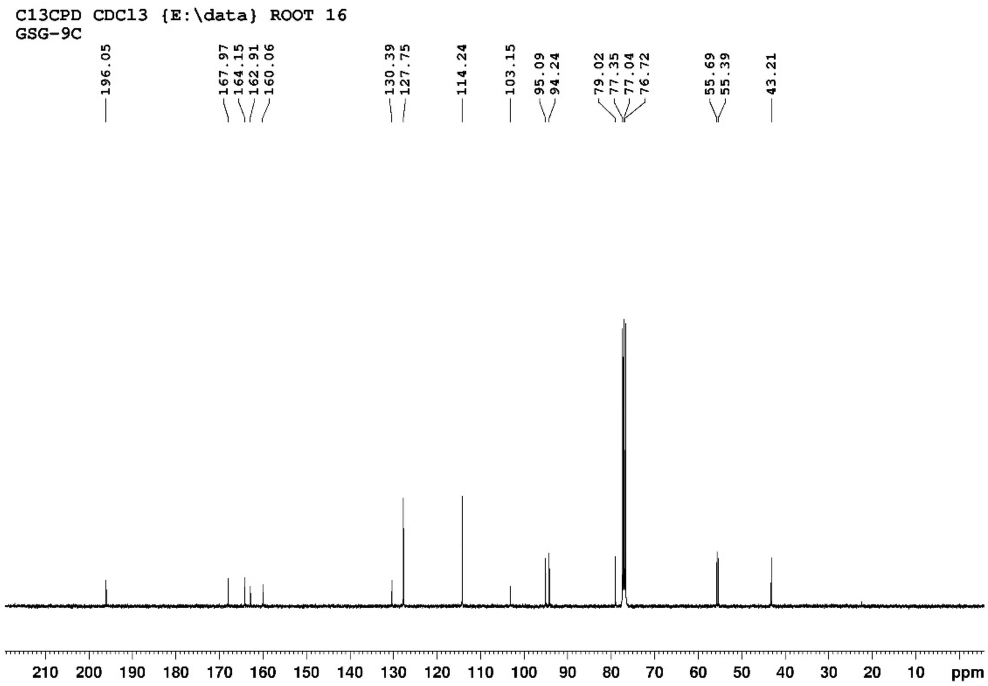

Figure S4. ^{13}C NMR spectrum of (−)-NRG-DM (100 MHz, $CDCl_3$)
